# Associação entre fadiga por compaixão e o perfil dos bombeiros que atuam em desastres

**DOI:** 10.1590/0102-311XPT230525

**Published:** 2026-07-27

**Authors:** Chancarlyne Vivian, Elisabete Maria das Neves Borges, Cristina Maria Leite Queirós, Vanessa da Silva Corralo, Samuel Spiegelberg Zuge, Maiara Dais Schoeninger, Letícia de Lima Trindade

**Affiliations:** 1 Universidade Comunitária da Região de Chapecó, Chapecó, Brasil.; 2 Escola Superior de Enfermagem, Universidade do Porto, Porto, Portugal.; 3 Faculdade de Psicologia e de Ciências da Educação, Universidade do Porto, Porto, Portugal.

**Keywords:** Saúde Ocupacional, Riscos Ocupacionais, Bombeiros, Occupational Health, Occupational Risks, Firefighters, Salud Laboral, Riesgos Laborales, Bomberos

## Abstract

O estudo analisa os níveis de fadiga por compaixão, satisfação por compaixão e *burnout* em bombeiros que atuam em desastres, e examina a sua associação com as características sociolaborais. Estudo misto, paralelo convergente com 555 bombeiros brasileiros. Na etapa quantitativa responderam a um questionário sociolaboral e ao *Professional Quality of Life Scale* ENT#091;Escala de Qualidade de Vida ProfissionalENT#093;, com achados submetidos à estatística descritiva e inferencial. Na etapa qualitativa 66 bombeiros responderam à entrevista e os resultados passaram por Análise Temática, sendo sequencialmente analisadas as inferências. Os participantes apresentaram maiores escores de satisfação por compaixão, seguidos pelos escores de *burnout* e estresse traumático secundário. A integração dos achados mostra como características sociolaborais e a atuação em contextos de desastres se relacionam a esses fenômenos, destacando-se a importância de reconhecer o perfil e os múltiplos fatores que podem impactar negativamente a saúde mental dos profissionais e/ou fortalecer as estratégias preventivas da fadiga por compaixão.

## Abstract (en)

This study analyzed compassion fatigue, compassion satisfaction, and burnout levels in firefighters who work in disasters and examined their association with socio-occupational characteristics. This mixed and convergent parallel study was carried out with 555 Brazilian firefighters. In its quantitative stage, participants answered a socio-occupational questionnaire and the *Professional Quality of Life Scale*. The findings were subjected to descriptive and inferential statistics. In its qualitative stage, 66 firefighters were interviewed. The results underwent Thematic Analysis, the inferences of which were sequentially analyzed. Participants had higher compassion satisfaction scores, followed by burnout and secondary traumatic stress scores. Integrating these findings shows how socio-occupational characteristics and work in disaster contexts are related to these phenomena, highlighting the importance of recognizing the profile and the multiple factors that can negatively impact the mental health of professionals and/or strengthen preventive strategies for compassion fatigue.

## Resumen

Este estudio analiza los niveles de fatiga por compasión, satisfacción por compasión y agotamiento en bomberos que trabajan en desastres y examina su relación con las características sociolaborales. Estudio mixto, paralelo y convergente con 555 bomberos brasileños. En la fase cuantitativa, los participantes respondieron un cuestionario sociolaboral y la *Professional Quality of Life Scale* ENT#091;Escala de Calidad de Vida ProfesionalENT#093;, y los hallazgos se sometieron a análisis estadístico descriptivo e inferencial. En la fase cualitativa, 66 bomberos respondieron a la entrevista y los resultados fueron sometidos a un Análisis Temático, analizándose las inferencias de forma secuencial. Los participantes reportaron puntuaciones más altas en satisfacción por compasión, seguidas de puntuaciones en agotamiento y estrés traumático secundario. La integración de los hallazgos muestra cómo las características sociolaborales y el desempeño en contextos de desastres están relacionados con estos fenómenos, destacando la importancia de reconocer el perfil y los múltiples factores que pueden afectar negativamente la salud mental de los profesionales o fortalecer las estrategias preventivas contra la fatiga por compasión.

## Introdução

A atuação de bombeiros em situações de desastres conduz a uma exposição contínua a eventos potencialmente traumáticos, em razão do intenso impacto emocional e psicológico dessas ocorrências e do papel que exercem ao prestar cuidados e apoio imediato às vítimas ^1^. A exposição contínua ao sofrimento humano constitui um importante fator de risco psicossocial para profissionais que atuam em contextos de emergência e resposta a desastres, o que expõe o trabalhador ao fenômeno da fadiga por compaixão, conceito introduzido por Charles Figley para descrever o desgaste emocional decorrente do envolvimento empático prolongado com o sofrimento de outras pessoas ^2^.

Apesar de amplamente investigada, a delimitação conceitual da fadiga por compaixão ainda apresenta desafios na literatura, frequentemente sendo confundida com os fenômenos isolados do estresse e do *burnout*. Estudos destacam que a fadiga por compaixão deve ser compreendida como um fenômeno complexo associado à exposição indireta a eventos traumáticos, resultante do processo de empatia e compaixão mobilizado no exercício profissional ^3^ ^,^ ^4^. O fenômeno também tem como aspecto definidor as experiências positivas relacionadas ao cuidado, como a satisfação por compaixão, que representa o sentimento de realização e propósito derivado da ajuda ao outro ^1^ ^,^ ^2^.

Assim, a fadiga por compaixão é uma síndrome que acomete profissionais de ajuda por atuarem diretamente com pessoas em situação de sofrimento ^2^ ^,^ ^5^. Trata-se de um fenômeno multidimensional composto por dois polos interligados: um polo positivo, denominado satisfação por compaixão, que se refere às experiências positivas associadas ao cuidado e ao sentimento de realização profissional, exercendo um papel restaurador na saúde psíquica; e um polo negativo, globalmente denominado por fadiga por compaixão, que engloba as vivências adversas decorrentes do trabalho de ajuda. Esse polo negativo subdivide-se em *burnout*, caracterizado por sintomas como exaustão emocional, frustração e irritabilidade, e estresse traumático secundário, relacionado a medos e traumas internalizados de forma indireta, a partir do contato empático com pessoas em sofrimento ^6^.

Nas últimas décadas, a intensificação e a gravidade dos desastres têm ampliado a demanda por competências de resposta entre profissionais que prestam cuidados às vítimas ^7^ ^,^ ^8^ ^,^ ^9^. Essa crescente exposição a situações de alta complexidade e sofrimento humano tem elevado o risco de adoecimento psicológico desses trabalhadores, especialmente daqueles que atuam na linha de frente das emergências ^10^.

Indubitavelmente, a atuação em contextos de desastres envolve elementos específicos para cada uma das tipologias do desastre, sejam eles naturais, tecnológicos ou causados por ações humanas. Esses cenários exigem preparo técnico, compreensão das dinâmicas socioterritoriais e integração intersetorial e interdisciplinar entre diferentes áreas do conhecimento e os diversos setores de resposta ^1^ ^,^ ^9^ ^,^ ^10^ ^,^ ^11^.

O processo de urbanização acelerada, aliado ao crescimento econômico desarticulado do planejamento territorial, à insuficiência de infraestrutura e à negligência em relação aos aspectos do ecossistema, configura um conjunto de fatores que contribuem para graves manifestações climáticas resultantes das crises socioambientais ^1^. Além disso, o aquecimento global intensifica eventos compostos, como ondas de calor e inundações, o que amplia os riscos à saúde. Esse cenário exige novas abordagens de gestão e ação. É fundamental identificar as cadeias de riscos, compreender os fatores que impulsionam os eventos extremos e a promoção de colaboração interdisciplinar, multirregional e intersetorial. Essas estratégias são essenciais para avaliar e mitigar riscos de forma eficaz, destacando a urgência de proteger profissionais e populações vulneráveis ^12^.

Exponencialmente, desastres afetam de maneira desproporcional as populações mais vulneráveis, exigindo ação coordenada para mitigar os danos e construir sociedades mais justas ^13^.

Notoriamente, estudos ^14^ ^,^ ^15^ realizados com profissionais de ajuda têm evidenciado associações significativas entre a fadiga por compaixão e determinadas características sociodemográficas e ocupacionais. Esses achados indicam que fatores como idade, tempo de experiência profissional, carga horária de trabalho e tipo de serviço prestado podem influenciar de maneira expressiva os níveis de fadiga por compaixão, revelando ainda, que diferentes perfis profissionais apresentam graus distintos de vulnerabilidades frente às demandas emocionais de cuidado.

A relevância do tema é evidente, uma vez que ainda há escassez de pesquisas nacionais e internacionais que investiguem de forma sistemática os fatores específicos associados à fadiga por compaixão em bombeiros, especialmente levando em conta as diferentes tipologias de desastres ^7^ ^,^ ^8^ ^,^ ^16^. Considerando a importância desses profissionais e de suas atuações, bem como a necessidade de preservar a sua integridade física e psicológica, torna-se fundamental a compreensão desses fatores a fim de subsidiar políticas institucionais, protocolos de prevenção e ações de apoio psicossocial coerentes com o contexto de atuação.

Diante disso, pretendeu-se analisar os níveis de fadiga por compaixão, satisfação por compaixão e *burnout* em bombeiros que atuam em desastres, e examinar sua associação com características sociolaborais.

## Método

Trata-se de um estudo de método misto, do tipo paralelo convergente ^17^, no qual a coleta e a análise dos dados quantitativos (QUAN) e qualitativos (QUAL) foram realizadas de forma concomitante e independente. Em seguida, os resultados provenientes de ambos os bancos de dados foram comparados e integrados, buscando convergências, divergências, integrações e expansão que pudessem oferecer uma compreensão mais abrangente ^18^ e aprofundada da atuação dos bombeiros em desastres. O estudo seguiu as recomendações das diretrizes *Strengthening the Reporting of Observational Studies in Epidemiology* (STROBE; Fortalecimento da Notificação de Estudos Observacionais em Epidemiologia).

O cenário do estudo compreendeu os estados brasileiros de Santa Catarina, Rio Grande do Sul, Rio de Janeiro e Minas Gerais, selecionados intencionalmente em razão do histórico recente de grandes desastres enfrentados nessas regiões. A escolha buscou contemplar diferentes realidades geográficas e socioambientais, permitindo uma análise comparativa mais ampla das condições de atuação e dos fatores de vulnerabilidade psicossocial dos bombeiros diante de eventos críticos.

Na etapa QUAN, foram convidados a participar bombeiros atuantes nos serviços operacional e administrativo em um dos cenários do estudo, com tempo mínimo de seis meses de exercício na função. Foram excluídos os profissionais afastados por qualquer motivo durante o período da coleta de dados.

Para a definição do tamanho amostral, considerou-se um nível de 95% de confiança e erro amostral de 5%, tomando como base um universo de 1.346 bombeiros. Dessa forma, a amostra mínima estimada foi de 300 participantes. Entretanto, todos os profissionais elegíveis foram convidados, resultando na adesão de 555 bombeiros ao estudo.

Na etapa QUAN, os participantes responderam a um questionário sociolaboral composto pelas variáveis: idade, estado civil, número de filhos, formação acadêmica, tempo de atuação profissional, tempo de serviço como bombeiro militar, jornada semanal de trabalho, cargo de ocupação e presença de outro vínculo empregatício. Além disso, foi aplicada a escala *Professional Quality of Life Scale* (PROQOL-5; Escala de Qualidade de Vida Profissional) ^3^, instrumento que avalia a qualidade de vida profissional de trabalhadores, composta por três dimensões: satisfação por compaixão, *burnout* e estresse traumático secundário.

Dos participantes que compuseram a etapa QUAN, 66 integraram a etapa QUAL, respondendo a uma entrevista semiestruturada guiada por roteiro com questões relacionadas ao trabalho e a vivências laborais. Os temas das entrevistas contemplaram vivências profissionais em desastres, com ênfase nas situações mais significativas enfrentadas, nos treinamentos realizados e nos efeitos emocionais e físicos associados ao trabalho.

Nessa etapa, a seleção dos participantes ocorreu por conveniência, ao considerar a disponibilidade para a entrevista. Apesar de a seleção ser por conveniência, destaca-se que foi assegurada a participação mínima de oito bombeiros por cenário, sendo adotado o critério de saturação teórica ^19^ como parâmetro para o encerramento das coletas, o que foi atingido na 55ª entrevista. Entretanto, mais 11 entrevistas foram realizadas com profissionais que expressaram interesse voluntário em contribuir com o estudo, totalizando 66 entrevistas analisadas.

A coleta de dados ocorreu entre fevereiro de 2024 e janeiro de 2025. Inicialmente foi realizado o contato prévio com as instituições participantes, apresentando os objetivos e procedimentos do estudo. Após o aceite institucional e a assinatura do Termo de Consentimento Livre e Esclarecido, os dados foram coletados presencialmente nos cenários de pesquisa ou mediados por tecnologia digital, sendo, além disso, as entrevistas audiogravadas mediante a autorização dos participantes e realizadas em ambientes privativos, assegurando confidencialidade, conforto e o sigilo das informações.

As análises QUAN foram realizadas no programa SPSS, versão 28.0 (https://www.ibm.com/). As variáveis contínuas foram descritas por média e desvio padrão ou, quando não apresentavam distribuição normal, por mediana e amplitude interquartílica. As variáveis categóricas foram apresentadas por meio de frequências absolutas e relativas. Para avaliar as associações entre as variáveis contínuas e ordinais, empregaram-se os testes de correlação de Pearson ou Spearman, conforme a distribuição dos dados. Adotou-se em todas as análises o nível de significância de 5% (p ≤ 0,05) e intervalo de 95% de confiança.

As análises QUAL foram conduzidas segundo a Análise Temática ^20^, envolvendo as etapas pré-análise, exploração do material e tratamento/interpretação dos dados, e apresentação da matriz categorial utilizada na análise, a qual contou com oito categorias temáticas, sendo utilizadas para responder ao objetivo deste manuscrito as categorias: (i) fatores que implicam a fadiga por compaixão e (ii) características profissionais que interferem na fadiga por compaixão.

Posteriormente, os resultados mistos foram integrados, seguindo os critérios de inferência, complementaridade e complexidade próprios da pesquisa de métodos mistos ^21^. Essa etapa permitiu a compreensão mais abrangente, profunda e contextualizada do fenômeno investigado, observando-se convergências e divergências ^22^.

O estudo atendeu às disposições da *Resolução nº 466/2012* do Conselho Nacional de Saúde, sendo apreciado e aprovado pelo Comitê de Ética em Pesquisa da Universidade Comunitária da Região de Chapecó (UNOCHAPECÓ; parecer nº 6.575.124). As entrevistas foram identificadas pela letra B (de bombeiros) seguida do número de ordem (B1, B2, ...), preservando o anonimato dos participantes.

## Resultados

Participaram do estudo 555 bombeiros, sendo 84,3% homens e 15,7% mulheres, com média de idades de 38 anos ± 8,6. A maioria declarou viver com companheira(o) (81,8%) e ter filhos (65,4%). Em relação à jornada laboral, 80% relataram atuar em turnos alternados nos três períodos do dia, sem um turno fixo. Quanto à categoria funcional, 11,9% dos bombeiros ocupavam cargos militares oficiais, 69% eram militares praças e 19,1% não militares.

A maioria dos bombeiros relatou atender frequentemente pessoas em sofrimento (84,8%) e em risco de vida (82,7%), bem como lidar com vítimas de eventos traumáticos (77,8%) e situações que os deixam emocionalmente impactados (55,3%). Apesar da exposição constante a contextos de alto estresse, os resultados indicaram elevada satisfação por compaixão (média = 40,3 ± 7,0), com quase metade dos participantes (48,8%) apresentando níveis altos, sugerindo realização e sentido positivo no trabalho. Em contrapartida, observou-se predominância de níveis baixos de *burnout* (66,8%) e de estresse traumático secundário (75,5%), o que aponta para um perfil de resiliência emocional e equilíbrio entre as demandas profissionais e o bem-estar psicológico (Tabela 1).

**Tabela 1:** Frequência de exposição a situações de sofrimento e médias dos domínios da *Professional Quality of Life Scale* entre os participantes (n = 555). Brasil, 2025.

Variáveis	Nunca n (%)	Raramente n (%)	Poucas vezes n (%)	Algumas vezes n (%)	Muitas vezes n (%)	Quase sempre n (%)
Você atende pessoas que estão em sofrimento?	2 (0,4)	38 (6,8)	44 (7,9)	149 (26,8)	178 (32,1)	144 (25,9)
Você atende pessoas em risco de vida?	6 (1,1)	40 (7,2)	50 (9,0)	188 (33,9)	191 (34,4)	80 (14,4)
Você atende pessoas que passaram por algum evento traumático? (p.ex.: abuso sexual, assalto, acidentes, agressão)	9 (1,6)	58 (10,5)	56 (10,1)	196 (35,3)	159 (28,6)	77 (13,9)
Alguns casos que você atende lhe deixam impressionado?	13 (2,3)	105 (18,9)	130 (23,4)	226 (40,7)	63 (11,4)	18 (3,2)
Domínios	Média	DP	Baixa n (%)	Moderada n (%)	Alta n (%)	
Satisfação por compaixão	40,3	7,0	17 (3,1)	267 (48,1)	271 (48,8)	
*Burnout*	19,9	6,9	371 (66,8)	181 (32,6)	3 (0,5)	
Estresse traumático secundário	17,0	8,3	419 (75,5)	133 (24,0)	3 (0,5)	

DP: desvio padrão.

A relação entre os domínios da fadiga por compaixão e as características sociolaborais indica correlações positivas entre o estresse traumático secundário e as variáveis idade, escolaridade, tempo de formação, tempo de atividade e jornada laboral, além de relações semelhantes entre *burnout* e escolaridade, tempo de atividade e carga horária semanal. Já a satisfação por compaixão apresentou correlação negativa com a jornada laboral, sugerindo menor satisfação em profissionais com maior carga de trabalho (Tabela 2).

**Tabela 2:** Relação entre os domínios da *Professional Quality of Life Scale* e as variáveis sociolaborais (n = 555). Brasil, 2025.

Variáveis	Satisfação por compaixão	*Burnout*	Estresse traumático secundário
Idade (anos)	r = 0,083	r = 0,025	r = 0,097 *
Número de filhos	r_s_ = 0,063	r_s_ = -0,001	r_s_ = 0,046
Escolaridade	r_s_ = -0,062	r_s_ = 0,132 **	r_s_ = 0,181 **
Tempo de formação profissional (anos)	r_s_ = 0,035	r_s_ = 0,080	r_s_ = 0,169 **
Tempo de atividade como bombeiro (em anos)	r_s_ = -0,050	r_s_ = 0,225 **	r_s_ = 0,267 **
Horas semanais de jornada laboral	r = -0,150 **	r = 0,263 **	r = 0,210 **

* p < 0,05;

** p < 0,01.

A integração dos achados mistos revelou uma compreensão ampla e integrada da vivência dos bombeiros frente às demandas emocionais do trabalho. Os achados indicam elevada exposição ao sofrimento humano, evidenciada por 32,1% dos participantes que relataram vivenciar essa situação “muitas vezes” e 25,9% “quase sempre”, corroborando depoimentos que descrevem o impacto emocional de lidar com tragédias e perdas. Apesar disso, a maioria demonstrou altos níveis de satisfação por compaixão (36,8% “muitas vezes” e 49% “quase sempre”), expressa nos relatos que associam o alívio do sofrimento alheio ao sentimento de realização pessoal e propósito profissional (Quadro 1).

**Quadro 1:** Triangulação dos dados quantitativos e qualitativos referentes aos domínios da *Professional Quality of Life Scale*. Brasil, 2025 (n = 555).

DOMÍNIOS	DADOS QUAN	DADOS QUAL
Local de trabalho	Atendem pessoas que estão em sofrimento 32,1% - Muitas vezes 25,9% - Quase sempre	“*Foi bem impactante, eu nunca tinha ido numa operação tão grande e pelo sofrimento que ainda tinha das pessoas que ficaram. Tinha muita gente que estava no dia da tragédia e sobreviveu, mas retornou lá para ajudar a encontrar os amigos, os familiares das pessoas*” (B11) “*Você vê o sofrimento, você impacta. Não tem jeito. A gente não é uma máquina. São situações que marcam*” (BM24) “*A gente vê muito sofrimento, não só pessoas mortas, mas pessoas que perderam tudo buscando alento, e às vezes, a gente é a única palavra de alento para essas pessoas*” (B37) “*Trabalhar com a pessoa em sofrimento,* (...) *porque você precisa fazer algo que no momento vai ser doloroso, desconfortável, estressante, mas que é necessário naquele momento*” (B43) “*A gente trabalha sempre na desgraça. Então é sempre no sofrimento das outras pessoas*” (B65)
Satisfação por compaixão	Sentem-se satisfeitos(as) por ser capaz de ajudar os outros 36,8% - Muitas vezes 49,0% - Quase sempre	“*A satisfação é ver que que o nosso trabalho é uma ação rápida, ela foi decisiva na vida dessas pessoas, isso é o mais satisfatório.* (...) *É uma satisfação imensurável você poder ajudar as pessoas e poder fazer a diferença na vida das pessoas*” (B1) “*Quando você faz um resgate a pessoa está em uma situação difícil e você dá uma qualidade para ela ter um atendimento. Quando você foi lá, combateu um incêndio e foi bem-sucedido, você amenizou aquele dano que poderia ser maior. Alguém poderia, por exemplo, ter perdido a vida e você conseguiu atuar e resolveu e apagou o incêndio, por exemplo, você tirou aquelas pessoas, dá uma satisfação imensa, quando tudo é bem-sucedido conforme o planejado*” (B4) “*O agradecimento da pessoa que a gente atende, é muito bom saber que a pessoa ficou feliz em ser bem atendida, em ser salva, isso para mim traz muita satisfação*” (B9)
*Burnout*	Sentem-se exaustos(as) por causa do trabalho 17,3% - Muitas vezes 28,8% - Algumas vezes 18,8% - Poucas vezes	“*Você vai de uma ocorrência e emenda na outra e eu já reparei que quanto mais tempo a gente fica privado de uma alimentação e de uma água, o estresse vai aumentando. Às vezes, você age com um pouquinho de agressividade, sem necessidade, porque às vezes, você está com fome, está com sede e não deu tempo de você parar*” (B9) “*Tinha uma amiga que tinha perdido um outro amigo, ele se suicidou usando fogo e ela não queria comentar a respeito. Na hora eu fiquei mais com vontade de fazer umas piadinhas, mas pensando depois eu vejo que como é uma situação que é grave, na verdade eu queria fazer mais piada do que sensibilizar. Eu me contive mais porque as pessoas não gostam. Então eu vejo em mim mais essa questão de desumanizar*” (B15) “*Eu estava no setor administrativo até esses dias, estava bem estressante. É frustrante porque lá realmente é estressante. Na instituição, os profissionais do setor administrativo eu vejo que são hoje os profissionais mais estressados, mais do que a atividade fim*” (B43) “*É como eu falei, a gente fica calejado, como eu falei no começo, a minha esposa diz que a gente é insensível. Então é um meio de defesa que a gente tem para não se envolver emocionalmente com as coisas, porque se não, não aguenta, não dá, a gente satura e não é pouco bombeiro que fica doente*” (B45)
Estresse traumático secundário	Recordam de partes importantes do trabalho com as vítimas do trauma 55,6% - Não recordam	“*Com o tempo, a gente aprende não absorver isso, a trabalhar melhor isso. No início da carreira, sim, isso me afetava mais. Hoje em dia nem tanto, não sei se o nosso subconsciente cria uma espécie de blindagem*” (B1) “*O fato de entender que é só uma profissão não tem o que fazer. Se a pessoa veio a falecer em uma ocorrência, pra mim ok. Eu sou apenas uma ferramenta de trabalho ali, que vai fazer algo que seja possível ali nesse cenário. Hoje eu sou um pouco mais frio nessa questão*” (B2) “*Inconscientemente, o nosso psicológico é afetado em algumas situações. Eu lembro de alguma ocorrência que não foi legal, um cadáver que estava com um odor muito forte. E aí, às vezes eu estou comendo, penso nisso às vezes, depois da ocorrência. Isso afeta, sabe? É um pouco ruim, porque cem por cento a gente não consegue desligar*” (B14) “*Tem vivências, por exemplo, se depara com um soterramento onde há a procura de uma criança, provavelmente morta, é uma ocorrência que marca*” (B18)

Em contrapartida, sinais de exaustão emocional foram observados em 17,3% “muitas vezes” e 28,8% “algumas vezes”, refletindo nas falas que evidenciam desgaste físico, estresse e estratégias de defesa como a insensibilidade e o distanciamento emocional. Por fim, a dimensão do estresse traumático secundário, com 55,6% dos profissionais relatando dificuldade em recordar partes de eventos críticos, revelou o impacto cumulativo das experiências extremas, embora alguns depoimentos indiquem mecanismos adaptativos e de resiliência desenvolvidos ao longo da carreira. Esses achados reforçam a complexidade emocional do trabalho dos bombeiros e a necessidade de políticas institucionais que promovam suporte psicológico contínuo e ambientes laborais mais saudáveis (Quadro 1).

Nos resultados QUAL, essa tendência foi reforçada por relatos que destacaram o estresse decorrente da rotina de atendimentos, o impacto emocional diante de situações de sofrimento e a sensação de exaustão diante de longas jornadas e experiências traumáticas. Observou-se ainda que os militares praças apresentaram maior probabilidade de contato com pessoas em sofrimento e com vítimas de eventos traumáticos, o que potencializa a vulnerabilidade à fadiga por compaixão. A convergência entre as evidências quantitativas e qualitativas demonstra que o cotidiano dos bombeiros militares é permeado por vivências emocionalmente intensas e recorrentes, que configuram um ambiente propício ao esgotamento e à fadiga emocional (Quadro 2).

**Quadro 2:** Integração dos dados sobre a fadiga por compaixão entre os participantes (n = 555). Brasil, 2025.

DADOS QUAN	CONCEITOS QUANTITATIVOS	INTEGRAÇÃO	CONCEITOS QUALITATIVOS	DADOS QUAL
Quanto maior a escolaridade (r_s_ = 0,132; p = 0,002) maior escore na subescala de *burnout*	Probabilidade de *burnout*	A escolaridade contribui para o polo negativo da fadiga por compaixão	As vivências negativas do trabalho são geradoras de esgotamento emocional	“*O estresse da ocorrência em si, a agitação em si, eu acho que tudo isso somando eu acredito que deve ter contribuído para todos esses sintomas*” (B9)
Quanto maior o tempo de exercício profissional (r_s_ = 0,225; p < 0,001) maior escore para *burnout*	Tempo de atividade como bombeiro	Os anos de atuação favorecem o esgotamento	Acúmulo de experiências profissionais propulsoras de sofrimento psíquico agudo	“*Se o grito de uma criança não está me sensibilizando, acho que está na hora de dar uma pausa porque está complicado. É uma coisa da atuação de muito tempo, e o cansaço do dia*” (B15)
Horas semanais de jornada laboral (r = 0,263; p < 0,001), favorecem maiores escores de *burnout*	Jornada laboral	O *burnout* aparece em bombeiros com longas jornadas de trabalho	Experiência subjetiva de cansaço extremo	“*A gente tem um tipo de escala que você fica aquartelado, tu não pode retornar para casa, tem que ficar à disposição do serviço. Trabalhava um dia 24 horas, folgava 24, só que essa folga, ficava em quartel*” (B63)
Os militares praças têm 33% maior probabilidade de atender pessoas que estão em sofrimento	Prevalência de fadiga por compaixão	A exposição contínua ao sofrimento gera vulnerabilidade elevada à fadiga por compaixão	O cotidiano profissional é permeado por experiências de sofrimento acumulado e desgaste emocional	“*Teu corpo às vezes não aguenta mais, mas você quer terminar aquilo ali, terminar com o sofrimento das pessoas em achar por familiares mesmo que sejam vivos, ou que tenham vindo a óbitos, a pessoa poder enterrá-la os seus parentes e acabar com aquele sofrimento*” (B38)
Militares praças têm 52% maior probabilidade de atender pessoas que passaram por evento traumático	Probabilidade de atendimento a vítimas de eventos traumáticos	Profissionais que atendem vítimas de eventos traumáticos apresentam maior propensão para fadiga por compaixão	Relatos de eventos traumáticos durante a carreira	“*Foi um evento traumático para todos, só caiu a ficha realmente que eu tinha perdido os dois colegas quando escutei a corneta, quando saiu os dois caixões que ali eu entendi, realmente não vai ter volta.* (...) *Por mais que nós sejamos profissionais, nós entramos em choque também, um evento traumático. Ainda mais que foi aqui no nosso círculo*” (B55)

## Discussão

Os resultados deste estudo evidenciam a complexidade emocional e psicológica que permeia a atuação dos bombeiros em situações de desastre, destacando a coexistência de aspectos positivos e negativos no exercício do cuidado. Observou-se que o aumento da carga horária semanal, do tempo de serviço e da exposição prolongada a eventos críticos se associam a níveis mais elevados de *burnout* e estresse traumático secundário, ao passo que reduzem a satisfação por compaixão. Esses achados reforçam a compreensão de que a rotina operacional, marcada por risco, sobrecarga e contato frequente com o sofrimento alheio, representa um fator de vulnerabilidade para o desenvolvimento da fadiga por compaixão. De forma convergente, os relatos qualitativos corroboraram esse cenário, revelando experiências de exaustão, sofrimento psíquico e necessidade de maior suporte institucional e psicossocial, o que ressalta a urgência de estratégias preventivas e de promoção da saúde mental entre esses profissionais.

Assim, percebe-se que a singularidade intrasferível da experiência da atuação dos bombeiros em desastres de grande magnitude, bem como a complexidade das mais variadas tipologias, tangenciam para eventos psicologicamente angustiantes e danos de ordem física, emocional, psicossocial e laborais, aumentando a vulnerabilidade dos profissionais à fadiga por compaixão ^5^ ^,^ ^7^. Descrita como um resultado, manifestado por comportamentos e respostas emocionais, decorrentes do contato com o sofrimento ou trauma experienciado por outra pessoa, a fadiga por compaixão, considerada um verdadeiro “custo do cuidado” ^5^ ^,^ ^23^, tem sido associada a características sociolaborais nas profissões de ajuda ^15^.

Estudos envolvendo profissionais que atuam em desastres têm mostrado como o cenário pode contribuir para o desenvolvimento de respostas somáticas ao longo do tempo ^24^, que perpassam desdobramentos de ordem física ^23^ e emocional ^24^ ^,^ ^25^. Esses agravos em saúde mental têm se manifestado como um fator de grande impacto nas corporações, especialmente em instituições caracterizadas por contínua exposição a situações de risco e estresse. Assim, aspectos relacionados com as características subjetivas, suporte pessoal, condições institucionais e complexidade das vivências laborais, influenciam de maneira significativa como o profissional lida com situações de estresse, experiências traumáticas, satisfação por compaixão e fadiga por compaixão.

Nesse sentido, as tendências estatísticas indicam que os militares praças (soldados, cabos, sargentos e subtenentes) têm maior probabilidade de atender pessoas que estão em sofrimento, quando comparados com militares oficiais (coronel, tenente-coronel, major, capitão, primeiro e segundo tenentes), o que pode favorecer o desenvolvimento de transtornos relacionados ao estresse ^26^ ^,^ ^27^.

A combinação dos dados denota que a maioria dos profissionais sentem-se satisfeitos por serem capazes de ajudar as pessoas e que a satisfação por compaixão se refere aos sentimentos de prazer que os profissionais experienciam a partir da sua atuação na prestação de cuidados, sendo considerada uma saída saudável diante de vivências da compaixão no trabalho ^6^. Adicionalmente, fatores como executar um bom trabalho e ter a atuação como um fator decisivo entre a vida e a morte de uma pessoa são elementos balizadores de satisfação para os bombeiros, uma vez que entendem que por meio de seu exercício conseguem fazer a diferença diante da missão que lhes foi conferida. A satisfação por compaixão representa sentimentos positivos derivados da capacidade de ajudar, bem como, de um cuidado competente, pode ser impulsionada pelo comportamento altruísta e pela sensação de realização e satisfação que os profissionais experimentam, sendo conhecida por ser um motivador do comprometimento com a profissão ^6^ ^,^ ^28^ ^,^ ^29^ e um fator de proteção importante para evitar a fadiga por compaixão ^15^.

Entre os profissionais do Corpo de Bombeiros, determinados perfis sociodemográficos e laborais - notadamente mulheres, indivíduos com filhos, aqueles que atuam em turnos noturnos, não militares e com múltiplos vínculos empregatícios - apresentaram escores mais elevados de satisfação por compaixão. Esses achados estão em consonância com estudos recentes, que identificaram associações significativas entre a satisfação por compaixão e variáveis como sexo (predominantemente feminino), demandas de trabalho e bem-estar ocupacional ^14^. De modo semelhante, outra investigação aponta que o relacionamento interpessoal, o reconhecimento profissional e as condições laborais mais favoráveis constituem fatores associados a níveis mais altos de satisfação por compaixão ^15^. Assim, os resultados obtidos corroboram a literatura ao sugerir que aspectos pessoais (como a parentalidade), características do vínculo empregatício (não militar e múltiplos vínculos) e a natureza do turno (noturno) influenciam positivamente o bem-estar derivado do cuidado compassivo. Tais fatores podem atuar como promotores da satisfação por compaixão, por favorecerem maior senso de propósito, responsabilidade social e equilíbrio entre as demandas e a experiência profissional, indicando que estratégias organizacionais pautadas no reconhecimento, no suporte institucional e na flexibilidade laboral podem potencializar a satisfação por compaixão entre profissionais de resgate e emergência.

Apesar de terem sido identificados baixos níveis de *burnout* entre os participantes, a fusão dos dados evidencia a presença da síndrome com destaque para a manifestação de cinismo, caracterizada indiferença e distanciamento emocional em relação ao trabalho, configurando uma forma de defesa psicológica frente à exposição prolongada a estressores ^30^ ^,^ ^31^. O cinismo é uma das três dimensões do *burnout* e se manifesta por meio da “insensibilidade”, além de sentimentos de negativismo ou ceticismo em relação às atividades profissionais, juntamente com a exaustão emocional e a sensação de ineficácia profissional ^26^ ^,^ ^32^ ^,^ ^33^. Nesse contexto, o profissional em *burnout* tende a adotar comportamentos rígidos e, em alguns casos, ignorar o sentimento de outra pessoa. Tal comportamento pode progredir para atitudes de hostilidade, afetando de maneira negativa a qualidade dos serviços prestados ^26^. Assim, maior escolaridade, tempo de exercício como bombeiros e horas semanais de jornada laboral foram estatisticamente significativos com os escores de *burnout*, o que é corroborado por outros estudos ^8^ ^,^ ^33^.

A integração dos dados endossa que a evitação é uma característica presente no meio bombeirístico, podendo ser propulsora de estresse traumático secundário. Além disso, há presença de esforços para evitar situações, atividades, pessoas, lugares, lembranças físicas, conversas ou situações interpessoais que despertem recordações do evento traumático ^24^ ^,^ ^34^. Somado a isso, os pensamentos intrusivos permanecem após a finalização do atendimento e surgem de maneiras e em momentos inesperados. Assim, as reações secundárias ao estresse traumático podem assemelhar-se aos sintomas observados em pessoas com estresse traumático secundário, manifestando-se por meio de revivescência, hipervigilância e comportamentos de evitação ^35^.

O estudo amplia a compreensão ao evidenciar que a atuação envolvendo crianças, assim como a perda de colegas por morte, parecem em diferentes países ^5^ ^,^ ^35^ ^,^ ^36^ ser situações favorecedoras do desenvolvimento do estresse traumático secundário. Sendo assim, o fenômeno pode ser um risco secundário na atuação em desastres, uma vez que profissionais nessas ocupações podem estar sob risco maior de desenvolver o fenômeno e sintomas que interferem no funcionamento diário das atividades profissionais ^24^ ^,^ ^35^ ^,^ ^36^.

Assim sendo, enchentes, desmoronamentos ^1^, incêndios ^37^, acidentes de trânsito e aéreos envolvendo grande número de vítimas ^38^ e rompimentos de barragens ^39^ ^,^ ^40^ têm sido cada vez mais frequentes mundialmente ^41^. A complexidade desses eventos demanda aos bombeiros habilidades operacionais e competências de gestão para atuar de maneira rápida e articulada. Desse modo, inúmeros são os riscos à saúde ^31^ ^,^ ^42^ dos profissionais, que não se limitam ao momento do desastre, mas que podem se manifestar como efeitos cumulativos a longo prazo ^27^ ^,^ ^43^.

Diante desses desafios, os profissionais devem permanecer em estado de vigilância tomando decisões críticas mesmo quando submetidos à pressão contínua de estressores psicológicos e ocupacionais. Essa pressão os expõe a um risco aumentado para agravos à saúde, sobretudo mental, incluindo irritabilidade, agressividade, impulsividade, frieza, ansiedade, tensão, choro, sintomas depressivos, *burnout*, distúrbios do sono e, em alguns casos, estresse psicológico que leva ao suicídio ^25^ ^,^ ^27^ ^,^ ^30^. Adicionalmente, lesões, espasmos musculares, alopecia, doenças autoimunes, hipertensão arterial sistêmica ^37^, leptospirose, hepatite e cardiopatia foram relatados pelos participantes como impactos na saúde física decorrentes do exercício profissional, sendo algumas dessas condições também descritas na literatura.

Para lidar com estressores relacionados com o trabalho, alguns profissionais utilizam estratégias de *coping* disfuncionais ^44^ como o consumo de álcool ^29^ e outras substâncias psicoativas ^25^, o que pode ser compreendido como um indicador de sofrimento psíquico e uma questão de saúde pública que demanda estratégias preventivas, programas de apoio psicossocial e políticas institucionais voltadas à promoção do bem-estar desses trabalhadores.

Uma revisão da literatura ^5^ indica que a compaixão é considerada um processo cognitivo, afetivo e comportamental que inclui cinco elementos: (i) reconhecer o sofrimento; (ii) compreender a totalidade do sofrimento na experiência humana; (iii) sentir empatia pela pessoa que sofre e se relacionar com a angústia (ressonância emocional); (iv) tolerar sentimentos desconfortáveis ​​que surgem em resposta à pessoa que sofre (por exemplo, angústia, raiva, medo) de modo a permanecer aberto e aceitar a pessoa que sofre; e (v) motivação para agir para aliviar o sofrimento. Assim, embora a compaixão seja uma característica essencial no desempenho da função dos bombeiros, quando exercida sem mecanismos adequados de regulação emocional pode tornar-se um fator de risco para a saúde mental dos profissionais.

Evidencia-se que a saúde mental relacionada com o trabalho apresenta caráter multidimensional, multifatorial e complexo, exigindo acompanhamento contínuo tanto por parte da instituição quanto da comunidade científica. Assim, é imprescindível pensar em estratégias que ofereçam cuidado não invasivo e empático para reduzir a fadiga por compaixão e fortalecer estratégias de enfrentamento dos profissionais. Os primeiros socorros psicológicos configuram uma abordagem colaborativa centrada na pessoa que adota postura empática para fomentar mecanismos de enfrentamento contextualizados e individualizados, constituindo-se como uma intervenção inicial destinada para os profissionais, a fim de proporcionar um espaço seguro para expressão sem imposição, com o intuito de discutir suas necessidades e avaliar qual é a melhor abordagem para lidar com a situação que está enfrentando ^45^.

O estudo tem limitações que devem ser consideradas na interpretação dos resultados, entre elas o delineamento transversal e a amostra, que inclui equipes com potencial treinamento para o enfrentamento dos desastres, sendo que não podem ser generalizados a outras equipes do país e requerem mais pesquisas em outros contextos socioculturais e países. Ainda, tem um viés típico dos estudos sobre saúde do trabalhador: não aborda os profissionais afastados no período da coleta de dados.

## Considerações finais

O estudo evidenciou níveis altos de satisfação por compaixão e baixos de *burnout* e estresse traumático secundário entre os participantes. Variáveis como escolaridade, tempo de atividade como bombeiro e horas de jornada laboral favorecem maiores escores de *burnout* que somadas a tempo de formação e idade potencializam o estresse traumático secundário. Também o cargo foi uma variável fortemente associada ao desfecho de estresse traumático secundário e *burnout*, uma vez que militares oficiais e praças apresentam aumentos em ambos os escores se comparados aos não militares. Somado a isso, características sociodemográficas (sexo e possuir filhos) e laborais (cargo, turno de trabalho, possuir outros vínculos) refletem maiores escores de satisfação por compaixão.

Os resultados revelam que os sentimentos de propósito, realização e prazer em ajudar outras pessoas constituem dimensões da satisfação por compaixão que atuam como fatores protetivos diante das adversidades inerentes ao trabalho desses profissionais. Em contrapartida, a manifestação do *burnout* expressa-se por meio do cinismo, traduzido em atitudes de indiferença e distanciamento emocional em relação ao trabalho, compreendidas como estratégias defensivas frente à exposição prolongada a estressores. Ademais, o estresse traumático secundário evidenciou-se pela presença de comportamentos de evitação e de pensamentos intrusivos recorrentes, refletindo dificuldades em elaborar experiências críticas e demonstrando a sobreposição entre o sofrimento emocional e o compromisso com a missão de servir. Esses resultados apontam para a complexa inter-relação entre o prazer de cuidar e o desgaste psíquico decorrente do enfrentamento contínuo de situações de riscos e de perdas.

A integração dos dados permite inferir que embora os participantes apresentem elevados níveis de satisfação por compaixão e baixos escores de *burnout* e estresse traumático secundário, determinados fatores individuais e laborais podem influenciar negativamente esses indicadores. A triangulação dos dados reforça a coexistência entre prazer e sofrimento no exercício profissional, ressaltando a satisfação por compaixão como um elemento protetivo frente ao desgaste psíquico decorrente da exposição prolongada a eventos potencialmente traumáticos.

Retoma-se a relevância da implantação de estratégias de cuidado voltadas para profissionais que atuam em contextos de desastres, contemplando programas de prevenção e capacitação, além de estratégias de suporte imediato, tanto em nível individual quanto coletivo e institucional, de modo a favorecer a promoção de ambientes de trabalho mais seguros e saudáveis.

## Data Availability

As fontes de informação utilizadas no estudo estão indicadas no corpo do artigo.
